# Liver and Gall Bladder

**Published:** 1894-06-30

**Authors:** 


### PROGRESS IN SURGERY.

### LIVER AND GALL BLADDER.

**Cholecystotomy.**—Ball and Bidwell report[1] a case of biliary colic, in which cholecystotomy was performed solely on account of sudden attacks of pain in the epigastrium; even the history of jaundice was not definite. There was absolutely no swelling, and very little tenderness in the region of the gall bladder, which could not be felt until the abdomen was opened; yet the gall bladder was closely packed with stones, without, however, any fluid. The operation was followed by recovery, and there were no more attacks of pain.

**Gangrenous Cholecystitis.**—Hotchkiss reports[2] this rare case. Sudden and severe abdominal pain began about six hours before the admission of the patient to hospital. There was no previous history of importance. Temperature 103 deg. to 104 deg. Fahr. Vomiting set in, and the next day abdominal section was performed. The gall bladder was found distended, about five inches long, and containing calculi. The lower end of the gall bladder was almost black, and apparently gangrenous. There was a good deal of peritonitis in the neighbourhood. It was found impossible to attach the gall bladder to the parietes as its walls were so friable. Vomiting continued, and death occurred seven hours after operation, or about thirty hours from the onset of the attack. One hour before death the temperature rose to 109 deg.

**Choledochotomy.**—Prof. Terrier performed[3] choledochotomy on a case in which he had previously performed cholecystectomy, in which bile flowed through the drain two days after operation. The jaundice subsided, but the fæcal matter remained colourless. The second operation was undertaken because the functional disturbances continued and the biliary fistula persisted. After having introduced into the fistula to the depth of 18 centimetres a sterilised catheter, which served as a conductor, he opened the abdomen in the median line, and found that a calculus existed behind the first portion of the duodenum. Cutting into the duodenum, he inserted the index finger, and discovered the calculus just above the ampulla of Vater. Another incision was made into the common bile duct, enabling him to extract the calculus, which was muriform and encysted, 18 millimetres in length and 16 in width. He thereupon practised retrograde catheterism of the bile ducts with a No. 10 catheter, sutured the duodenum first with double rows, and then the common bile duct, and closed the exterior wound in applying a drain. No accidents followed the operation, and the patient made a rapid recovery.

**Cholecystenterostomy.**—Murphy records[4] seventeen successful cases by means of his anastomosis button. He uses a modification of this method, known as the "button tube," for cases where the condition of the gall bladder, on account of gangrene of its walls or extensive adhesions all around it, renders it impossible to either suture it to the abdominal wall or approximate it to any portion of the intestinal tract, and where it is compulsory to secure drainage of the bladder, or to use the bladder as a canal to allow the escape of bile in obstructive jaundice that jeopardises the life of the patient. This "button tube drainage" of the pattern here presented has the following advantages: (1) It can be easily and rapidly inserted deep in the abdominal cavity, though the gall bladder may be very much contracted; (2) it prevents with certainty the contact of the gall bladder contents with the abdominal viscera until such time as adhesions have formed around the tube; and (3) it leaves a large opening, when the instrument is withdrawn from the gall bladder, through which calculi can be extracted. The operation with the "button tube" is performed as follows: An incision is made in the abdominal wall in the usual position for operations on the gall bladder, beginning at the ninth costal cartilage, parallel to the external border of the rectus muscle for a distance of two and a half inches. The gall bladder is located, a sufficient surface of its wall exposed, the contents aspirated, the purse string suture inserted, the gall bladder incised, male half of button inserted, purse string tied and cut short; the tubular portion of the button is then pressed into position; the tube is then drawn out as far as the gall bladder will permit, and held there with a pin passed through the openings in the side. During the time the pressure atrophy in the portion of the gall bladder clasped between the button is taking place, a cicatricial wall is being formed about the tube, which acts as the walls of a sinus after its production, and insures continued protection to the peritoneal cavity.

In comparing[5] the results of the ordinary operation of cholecystotomy with cholecysenterostomy Dr. Weir states that all patients died when the entire quantity of bile secreted escaped through the fistula, and where a large proportion escaped the patients became emaciated and sick. It is, therefore, desirable to have a safe means of allowing the bile to re-enter the intestine. There are reported in all, up to December, 1893, 23 cases operated on in one sitting, all by means of sutures, with a mortality of 35 per cent., or 8 deaths in 23 cases. From June 11th, 1892, to December 1st, 1893, there were 17 cases of cholecysenterostomy operated on for gall stones by means of the anastomosis button, with 17 recoveries—100 per cent. This latter operation is indicated (1) in all cases where it is desirable to drain the gall bladder for accumulations therein; (2) in all cases of cholelithiasis where obstruction of common duct is present, or where the reflex disturbances of digestion are marked; (3) in all cases of perforation of the choledochus into the abdominal cavity, where the duct must be obliterated by reparative process; (4) in all cases of cholecystitis, either with or without gall stones; (5) in all profusely discharging biliary fistulæ, either following operations or as sequelæ of pathological changes in the gall tract. The cases in which this operation should not be performed are: (1) All cases where the gall bladder is too small for the insertion of the button; (2) where the adhesions are so extensive that the bowel cannot be brought into contact with the gall bladder without kinking; (3) where the ductus cysticus is obliterated, in which cases the operation of cholecystectomy should be performed where there is an absence of adhesions around the gall bladder; (4) where we have an enormously enlarged gall bladder, with an elongation of the ductus cysticus, and without an obstruction of the ductus choledochus, in which case cholecystectomy should be performed. If the ductus choledochus be occluded, the gall bladder should be amputated just above the neck, leaving a sufficient portion in which the button can be inserted in the end and the approximation made to the duodenum in the usual way.

Dr. Murphy thinks[6] that the classes of cases requiring surgical interference in lesions of the gall tracts are as follows: (1) In all cases of obstructive jaundice, where the jaundice exists during a period of two weeks; (2) in all recurrent cases of obstructive jaundice; (3) in cases of recurrent cholecystitis; (4) in recurrent cases of colic from cystic duct obstruction, always without jaundice; (5) in retention cysts of gall bladder; (6) in malignant growths of gall bladder. Early operation is the most favourable, as the gall bladder is not contracted and is free from adhesions.

**Hydatids of the Liver.**—Tyson reports[7] a case of suppurating hydatid cyst of the liver, opened through the chest wall at the eighth interspace. The drainage tube was not finally removed for more than a year after operation.

[1] Lancet, Mar. 10th, 1894, p. 600. [2] Annals of Surgery, Feb., 1894. [3] Medical Week, March 9th, 1894, p. 114. [4] Medical Record, Jan. 13th, 1894, pp. 35 it. seq. [5] Op. Cit. [6] Medical Record, Jan. 20th, 1894, p. 69, &c. [7] Brit. Med. Journ., Jan. 20th, 1894.

